# What matters most in acute care: an interview study with older people living with frailty

**DOI:** 10.1186/s12877-022-02798-x

**Published:** 2022-02-25

**Authors:** James David van Oppen, Timothy John Coats, Simon Paul Conroy, Jagruti Lalseta, Kay Phelps, Emma Regen, Peter Riley, Jose Maria Valderas, Nicola Mackintosh

**Affiliations:** 1 Department of Health Sciences, University of Leicester, Leicester, UK; 2 Emergency & Specialist Medicine, University Hospitals Leicester NHS Trust, Leicester, UK; 3 Department of Cardiovascular Sciences, University of Leicester, Leicester, UK; 4 MRC Unit for Lifelong Health and Ageing, University College London, London, UK; 5 Leicester, Leicestershire and Rutland Older Persons Patient and Public Involvement Forum, Leicester, UK; 6 Department of Family Medicine, National University Health System, Singapore, Singapore

**Keywords:** Frailty, Geriatrics, Emergency medicine, Person-centred care, Patient satisfaction

## Abstract

**Background:**

Healthcare outcome goals are central to person-centred acute care, however evidence among older people is scarce. Older people who are living with frailty have distinct requirements for healthcare delivery and have distinct risk for adverse outcomes from healthcare. There is insufficient evidence for whether those living with frailty also have distinct healthcare outcome goals. This study explored the nature of acute care outcome goals in people living with frailty.

**Methods:**

Healthcare outcome goals were explored using semi-structured patient interviews. Participants aged over 65 with Clinical Frailty Score 5-8 (mild to very severe frailty) were recruited during their first 72 hours in a UK hospital. Purposive, maximum variation sampling was guided by lay partners from a Patient and Public Involvement Forum specialising in ageing-related research. Qualitative analysis used a blended approach based on framework and constant comparative methodologies for the identification of themes. Findings were validated through triangulation with participant, lay partner, and technical expert review.

**Results:**

The 22 participants were aged 71 to 98 and had mild to very severe frailty. One quarter were living with dementia. Most participants had reflected on their situation and considered their outcome goals. Theme categories (and corresponding sub-categories) were ‘Autonomy’ (information, control, and security) and ‘Functioning’ (physical, psychosocial, and relief). A novel ‘security’ theme was identified, whereby participants sought to feel safe in their usual living place and with their health problems. Those living with milder frailty were concerned to maintain ability to support loved ones, while those living with most severe frailty were concerned about burdening others.

**Conclusions:**

Outcome goals for acute care among older participants living with frailty were influenced by the insecurity of their situation and fear of deterioration. Patients may be supported to feel safe and in control through appropriate information provision and functional support.

## Introduction

As emergency departments (ED) worldwide become crowded increasingly frequently, poorer attainment of time targets can be expected [1]. In Europe, around one tenth of ED patients are older people living with frailty [2]. Older people living with frailty are at particular risk of prolonged length of stay, as well as other adverse outcomes including mortality and functional decline [3]. These outcomes are service metrics and, while useful for managers, may not capture what older people consider meaningful [4].

### Frailty

Frailty is a physiological syndrome causing vulnerability to adverse outcomes from apparently minor stressor events [5]. It helps explain variability in ageing, and why individuals of the same age experience different health and outcomes: as more physical, psychological, and social deficits are accumulated, physiological responses are progressively impaired [6]. Although associated with ageing and more common in older populations, most older people are not frail, and therefore frailty needs to be ascertained on an individual basis.

The Clinical Frailty Scale (CFS) uses pictorial and descriptive representations of people with progressive degrees of frailty, and has been widely adopted in NHS acute care settings owing to its prognostic properties and ease of application [7–9].

People who have frailty are best served by multidisciplinary, holistic care [10]. Various models can be applied to structure the delivery of such care. There is robust evidence supporting the Comprehensive Geriatric Assessment (CGA) approach to reduce mortality and institutionalisation [11]. Using CGA, healthcare professionals and patients develop an personalised problem list and management plan. The ‘4Ms Framework’ is grounded in the same principles and considers each older person holistically in terms of mobility, mentation, medication, and what matters [12]. The latter item is professionals knowing and aligning care with each older person’s healthcare outcome goals.

### Healthcare outcome goals

Healthcare outcome goals can be defined in terms of selected outcomes of treatment or aspects of health status. Healthcare preferences are the processes a person is willing to undergo in order to achieve their outcome goals [13]. Existing service metrics cannot capture the complexity of healthcare outcome goal or preference attainment. Standardised questionnaire instruments have been developed to enable comparison of patients’ perspectives on their outcomes (Patient-Reported Outcome Measures – PROMs) and experiences (Patient-Reported Experience Measures – PREMs) [14].

A recent systematic review of evidence reported older people’s outcome goals for acute care to be not limited to timeliness, but also to include receiving holistic, sensitive, and informed care [15]. The review reported a lack of evidence enabling stratification based on frailty. Indeed, most research has selected on the basis of age only rather than investigating for the effect of frailty [16].

Currently, older people’s healthcare outcome goals are often not discussed or achieved in hospital [17]. In this study, we aimed to build on previous research and develop a more nuanced understanding of older people’s acute healthcare outcome goals. People living with frailty have distinct perspectives and life experiences [18]. This study aimed to describe their outcome goals for acute care.

## Methods

### Study design and setting

This study sought in-depth accounts of people’s perspectives on healthcare outcome goals. An interpretive approach was therefore taken, involving interviews with older people who were living with frailty. Participants with current or recent experience of requiring emergency and acute healthcare were recruited.

Patient participants were recruited from a single, large National Health Service hospital located centrally in the UK, with a catchment population of 1 million encompassing one of the country’s most culturally diverse cities. The ED receives over 250,000 annual attendances, with around one in ten patients living with frailty [8]. Potential participants were recruited during attendance at the ED or within the first 72 hours of admission to an acute care ward.

### Selection of participants

Patients aged over 65 with a Clinical Frailty Score 5 to 8 (‘mild frailty’ to ‘very severe frailty’) were eligible [7]. At the recruiting ED, the CFS is administered routinely by trained triage nurses and displayed on departmental dashboard software. The CFS score was prefixed ‘D’ for people with frailty who had a software alert that they were living with a diagnosis of Major Neurocognitive Disorder (‘dementia’). The CFS was confirmed by the researcher at the time of recruitment. Informed, written consent was recorded, either by participants themselves or by consultees where they did not have capacity to consent.

The purposive, maximum variation sampling strategy was developed with lay research partners from a Patient and Public Involvement (PPI) Forum focused on ageing-related research. Lay partners were aged 65 or older and living with frailty or had experience caring for others with frailty. During the recruitment process, participants’ demographics were discussed with the lay partners so that under-represented population groups could be identified. We aimed to recruit a diverse sample which broadly represented our local city population. We did *not* exclude potential participants based on their presenting concern or on medical history such as cognitive impairment. We recruited face-to-face on weekdays, weekends, and public holidays, and at both day and night. Recruitment continued until additional data no longer yielded new themes [19, 20].

### Interviews

Interviews were arranged at participants’ convenience within one month of their acute care episode, with the first author (a male, early-career emergency physician and PhD candidate). Initially, interviews took place in participants’ homes. During COVID-19 restrictions, interviews took place whilst participants were still in hospital or by telephone/video-call after discharge. There was no prior relationship between participants and the researcher, who explained the objectives using information sheets.

In order to help to overcome potential barriers to communication, including cognitive impairment and language, patient participants were interviewed with their relative or familiar caregiver if they so wished. Those relatives and familiar caregivers who were interviewed, consented to participate. The interviewer focussed on the views of the patient participant rather than those of people accompanying them. Those accompanying helped with prompting recall for participants with cognitive impairment who could express opinion but not detail, for instance, or with clarifying points of expression for participants with limited English. We did not ask accompanying participants to translate sensitive topics and we did not interview relatives or caregivers alone.

Interviews were semi-structured and used a topic guide (Appendix 1) which had been developed from literature review themes and piloted with lay partners. Audio-recorded interviews sought to ascertain health outcome goals by exploring participants’ reflections upon their situation, concerns, health needs, and preferred treatments. The interviewer’s reflections were captured in field notes, written after each interview.

### Analysis

Data analysis used a blended approach based on constant comparison and framework methods and was simultaneous with collection. Audio recordings were transcribed verbatim and anonymised by an external professional contractor. The recordings were listened to by the interviewer and the transcripts checked for accuracy. The transcripts were then read, re-read, and reflected upon individually and later collaboratively by the first author and two research fellows. Reflection comments were annotated alongside transcripts and were used to inform assigning initial open code identifiers to aid navigating the data [21]. The dataset was managed using NVivo software [22].

The starting point for organising a framework of themes was a systematic review of evidence for emergency care outcome goals among a general population of older people (both having and not having frailty) [15]. Transcript evidence, or absence thereof, supporting *a priori* themes derived from the literature review (comprehensive, person-centred care, vulnerability, and information provision) was sought, and mapped with corresponding data codes. Iterative revisions were informed by seeking contradictory and absent evidence [23]. Substantive codes for new themes were developed inductively. The emerging framework themes were constantly iterated, merged, and compared back to earlier data as new interviews were collected [24, 25]. The process aimed to remain open to identifying theoretical connections between themes [26].

The effects of relationships between interviewer, participant, environment, and data were constantly reflected upon during data collection, review of recordings and transcripts, data coding, and collation of findings. Instances where data may have been influenced by the identity, relationship, and position of the researcher were recorded using separate code identifiers. These were regularly reviewed and discussed among the research team. The COREQ checklist was used to support consistency of reporting [27].

### Validation

Triangulation approaches were used to judge validity and inform iterations to the emerging framework. Respondent validation involved showing and discussing printed drafts of progressive framework summaries with participants in later interviews: participants were asked how well the themes represented their goals, and specifically what was missing or presented incorrectly.

Analyses were shared for discussion and iteration with lay research partners from the specialised PPI Forum. A technical panel was also consulted, formed of qualitative methodologists (two), healthcare professionals (six), and service managers (three) in acute geriatrics and emergency medicine from Canada, Netherlands, and United Kingdom. Meetings with lay research partners and technical consultants were conducted in-person, by video-call, and by email. Discussions were around improving the relevance, appropriateness, comprehensiveness, and meaningfulness of categories. In particular, the lay research partners identified topics for further enquiry and ensured that analyses and presentation remained grounded in the patient voice.

## Results

### Characteristics of study participants

Meaning saturation of themes was reached with 22 interviews. Participants’ characteristics are summarised in Table 1; the mean was age 85 years (range 71 to 98) and frailty ranged from mild to very severe (CFS 5 to 8). Most participants (*n*=19) were interviewed alone, and three quarters (*n*=17) used English as their first language. One quarter (*n*=5) were living with dementia. Interviews lasted 18 to 74 minutes.

**Table 1:** Summary characteristics of study participants

Variables	Participants (Total: 22)
**Age group (years)**	
65-74	3
75-84	7
85-94	10
95+	2
**Clinical frailty score**	
5	3
6	12
7	6
8	1
**Cognitive impairment**	
Living with dementia	5
**Sex**	
Female	15
Male	7
**Ethnic group**	
Asian	5
Black	1
White	16
* Having limited or no English* * Using English as additional language*	2 3
**Living arrangement**	
Living in own home	21
* Of those living in own home:*	
Living alone Living with partner Living with other generations Receiving social care	11 8 2 12
Living in residential care	1
**Interview location**	
Own home	3
Telephone	1
Hospital	18
**Interview participants**	
Alone	19
Accompanied by relative	3

### Main results

**Fig. 1:**
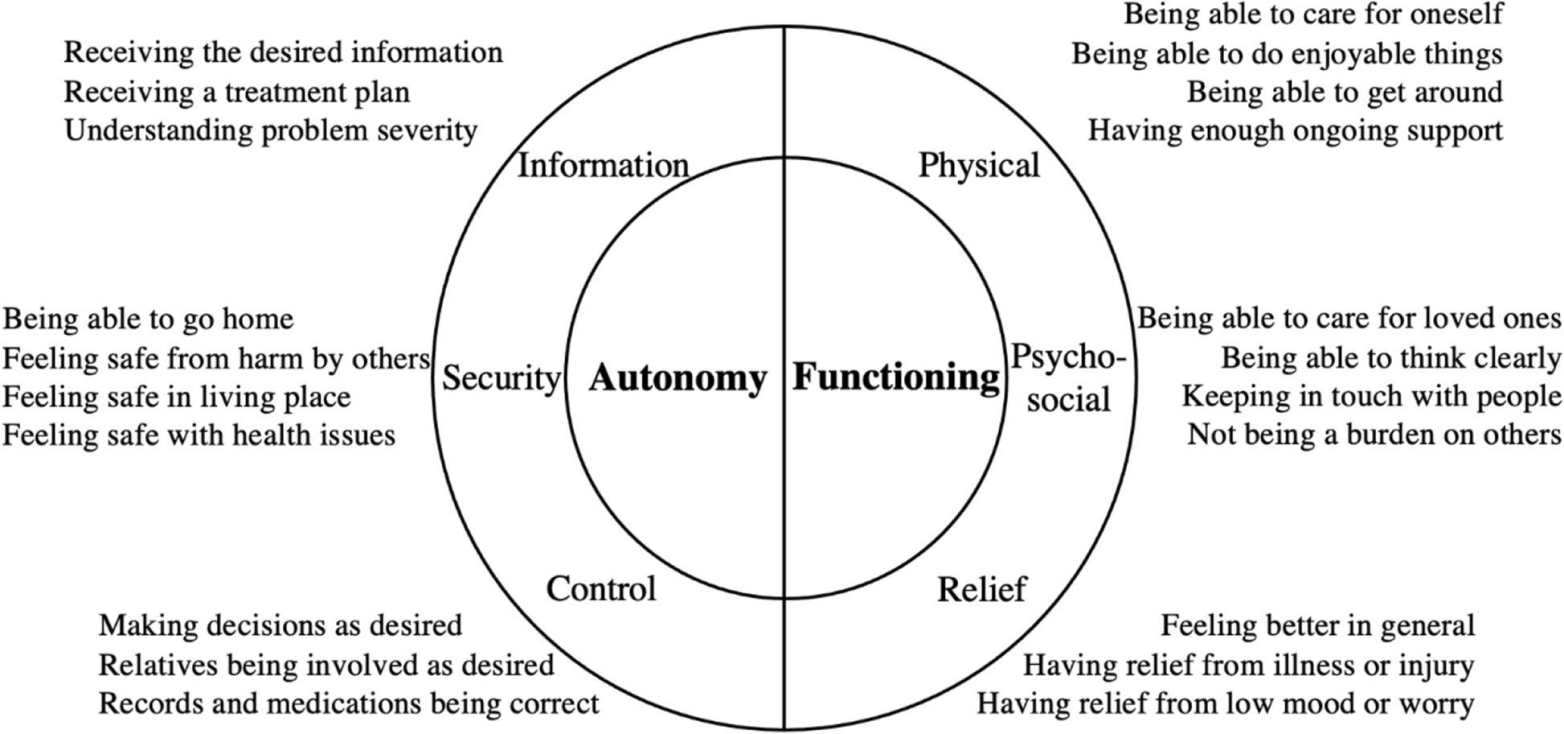
Framework of acute healthcare outcome goals among older people living with frailty

Most participants were able to express specific outcome goals, having attended the ED with these in mind. Core categories of Autonomy and Functioning were subcategorised respectively as ‘information, control, and security’ and ‘physical, psychosocial, and relief’ (Fig. 1). A substantive proportion of the dataset is presented to illustrate all higher-level domains and most sub-themes; in the interests of brevity, not all data is presented. Many topics were closely related and therefore the margins between categories were indistinct. One theme suggested by a lay research partner (feeling safe from harm by others) lacked interview evidence but was included with unanimous support from the PPI Forum.

### Autonomy

#### Information

##### Receiving the desired information

Participants had substantial life experience and were very familiar with their diagnoses, having adjusted to living with multiple problems. They needed to feel heard and understood, and to have their life and health experience acknowledged. They expected clear communication with active listening and wanted to be the focus of professionals’ attention rather than their notes or computers. Participants were frustrated when their information goals were unmet, but often tolerated this perhaps through empathy with busy staff:

*It seems that questions are left unanswered. They're so overworked though you could understand it. Not being able to spend as much time with you as you like but there you go. (Participant 6, CFS 6)*

##### Understanding problem severity

People wanted to know what problem was causing their symptoms and wanted to leave the department with a clear plan for its rectification. They were concerned about their symptoms, and sought reassurance through understanding their problem’s potential trajectory:

*I knew things weren’t right, but I didn’t know what was wrong. I have had sepsis and they thought they were going to lose me, and since then I’ve always been a bit frightened when I haven’t been well. I just wanted somebody to reassure me that everything would be all right. (Participant 18, CFS 6)*

#### Security

##### *Feeling safe with health problems* and *Feeling safe in living place*

Nine participants were living without professional care support, and were afraid of becoming unable to manage their daily routine. They feared accidents around the house attributed to ‘clumsiness’ or ‘forgetfulness’. Participants sought to feel safe at home, through help from others or through adaptations to their routines:

*My fear is that I can’t get there quick enough when things go wrong, because I can’t move, and that frightens me. I fry my bacon now, I don’t grill it – because, well I don’t want to set that on fire again. Fire frightens me more than anything else. (Participant 1, CFS 6)*

Participants frequently had firm insight into their frailty, knowing themselves to be vulnerable to further health problems. They were aware of their deteriorating health and increasing dependence on others and were afraid of sudden illness following previous experience affecting themselves or loved ones. Those with more advanced age or greater degree of frailty shared existential concerns:

*She does worry about what’s going to happen because she always thinks that she’s going to die soon. What’s going to happen to me? How is that going to happen? Will I be in pain? Will God take me to a good place or a bad place? (Participant 21, CFS D7, relative translating)*

##### Being able to stay at home

Despite concerns around physical safety, participants generally regarded their home environment as sanctuary, and hoped to stay at home rather than be admitted to hospital. Nearly all participants had been reluctant to attend the hospital, and some had consciously opted to tolerate symptoms and delay seeking help. Some participants had tried to ‘sleep off’ their problem in the hope that symptoms would resolve, fearing the unfamiliar processes and places at hospital or the potential diagnoses they might receive:

*It felt as if I were going to pass out and collapse. I thought I were going to fall. I thought oh, I shall perhaps be all right in the morning. I thought I might sleep it off and feel a bit better. But I didn’t. I wasn’t all right. (Participant 7, CFS 5)*

##### Feeling safe from harm by others

This theme was included based on lay partner advice that affected people may not feel able or safe to discuss topics relating to abuse with researchers or healthcare professionals. Phrasing aimed to have resonance for those who may fear harm from both familiar and unfamiliar people.

*My concerns are mostly for people who are not in such a supportive home, who live alone or who are overwhelmed by their situation: I can think of many reasons why someone would not answer freely. (PPI review round 7, emailed comments)*

#### Control

##### Making decisions as desired

Many participants’ information requirements related to their need to participate in decisions about the diagnosis and management of their problem. Most wished to take an active role in their healthcare and to be involved in decision-making or choices about transfer or treatment options.

*I knew I would have the basic examination. And I thought I might want a brain scan and then talk about the official diagnosis. (Participant 3, CFS 6)*

Some participants preferred important decisions to be made by their clinical team or their relatives, but still wanted to be kept informed about decision outcomes and what would happen next. This was perhaps due to them feeling pressured to make choices or concerned not to make mistakes. Participants were often cognisant of the risks and potential complications associated with healthcare treatments:

*They said ‘how do you feel about going to the hospital?’ So I said, ‘I’m no good as I am at home’. I was a bit apprehensive to come in here because of the Covid business but thought well, if I don’t, I’ve got to wait till tomorrow to ring the doctor’s and then it could happen again. (Participant 7, CFS 5)*

Some participants were frustrated when they felt professionals were withholding information, whereas others did not wish to know the full clinical details. They observed the importance of tailoring the consultation around individual preferences:

*You’ll only have confidence in professionals if they are honest with you. Lying by omission is just as bad as telling a straightforward lie, you know. The option for blunt information should be there … I don’t think everybody is of a mind that can accept it, but I think the option should be given. (Participant 13, CFS 6)*

##### Relatives being involved as desired

Many participants received support from friends, neighbours, or relatives. Some felt it important that these ‘important others’ were kept updated in all aspects of healthcare and may have deferred decision-making to their partners or children. Others did not want their important others ‘bothered’ unless there was crucial information, and some did not want them contacted at all. Involving important others in consultations often helped to overcome communication barriers:

*Of course it is important to contact your relatives. You are worried you will lose control. I wish people spoke the language I speak. I find it hard to tell people what’s going on. (Participant 20, CFS 7, relative translating)*

##### Records and medications being correct

Participants reported feeling insecure when their routines were changed to conform with system constraints, perceived as ‘rules’. Medications were a frequent source of worry, particularly when participants did not understand the rationale for alterations. They were frustrated when information gathering was duplicated. There was an assumption that computer systems enabled seamless information transfer between clinicians, and annoyance when communication proved inefficient:

*The nurse who spoke to me from the ambulance service had said I needed to go to hospital, so I’d got my bag and tablets ready by the time they got here. But I had to repeat the whole story of what had happened and everything, and they said no we won’t take you. (Participant 18, CFS 6)*

### Functioning

#### Physical

##### Being able to care for oneself

Many interview participants were living alone in their own homes and had learnt to adapt to deficits in function. Hospital attendances were often triggered by sudden and sometimes small changes in health, living environment, or routine. Participants needed to maintain their independence:

*Even though I walked slowly, at least I was able to do things for myself. Whatever little that is, at least it gives me a little bit of independence. Now I cannot stand up or do anything for myself. Not been able to shower, not been able to go to the loo. So, I want to get back on my feet. I want that to return to normal at least. (Participant 20, CFS 7, relative translating)*

#### Being able to get around

Independence goals persisted even once participants’ frailty had progressed to them developing dependency on relatives or carers. They were aware of the effects of relatively minor problems contributing to poor global function, and used these insights to form specific outcome goals:

*I need a frame to be able to get out of the hospital. I have got to be able to walk. (Participant 2, CFS 5)*

#### Having enough ongoing support

The discharge preparation phase posed a significant worry for many participants, who felt unsure of timeframes towards discharge or whether they felt safe to go home. Many participants had increased care needs due to gradual or sudden inability to manage their daily routines. They expected information to be passed to their primary care professionals, and wanted follow-up appointments and visits to be arranged:

*Obviously they’ve passed it on – I’m going to see the physio tomorrow at the community hospital, to assess me, and see what they can do to help me. Whether I’ll need exercise or manipulation or whatever. (Participant 2, CFS 5)*

#### Psychosocial

##### *Being able to care for loved ones* and *Not being a burden on others*

Participants were concerned about the effect of their situation on other people. Those living with lesser burdens of frailty (CFS 5-6) were often concerned to maintain independence and ability to care for others whether for their partner or pet. Their loved ones often depended on them, with their welfare presenting a barrier to participants seeking emergency care themselves.

*If they say to me that you’re staying overnight, the wife’s going to be struggling tonight. She can’t lift the kettle to pour a cup of tea, you know. I mean she’s in the kitchen and I’m sat in the lounge and she’ll call me to pour her a cup of tea because she can’t do it. (Participant 7, CFS 5)*

Those with more severe frailty (CFS 7-8) were often concerned about burdening their loved ones:

*I still prefer it at home, but what’s the point of going home in this state when I’m a headache to myself and everyone else? Everyone is so worried about me; I’d rather get better than go home. (Participant 20, CFS 7, relative translating)*

#### Relief

##### *Having relief from illness or injury* and *Feeling better in general*

Many participants believed that their chronic illness or disability would not improve. As well as often having severe pain, participants experienced symptoms which were different to characterise and describe, causing them to feel ‘generally unwell’. Some were unsure what was happening to them or what treatments might be possible. They attended wanting ‘to feel better’:

*I don’t know what’s causing it. I don’t know what they can do now, to be honest. I feel as if I need something to take it away. Whether it’s some medication or something, I don’t know. (Participant 7, CFS 5)*

##### Having relief from low mood or worry

Some participants suffered with mental health symptoms which affected their wellbeing and interactions with others. Others were worried about their general health or the potential outcomes from their acute problem:

*[Translator] I know that I am going to die eventually. I am getting to that age. But I still worry about it. Some days it can make me feel a bit down. Then it can affect my sleep as well, and that can affect my eating as well. (Participant 21, CFS D7)*

## Discussion

To our knowledge, this is the first study of older people’s acute healthcare outcome goals including a specific consideration of frailty. This population is distinct in terms of their outcomes from healthcare and the system models they require in the acute setting [3, 28]. This study has identified that there are also distinct aspects to the healthcare outcome goals which people with frailty consider to be important and meaningful. Participants had often spent substantial time reflecting on their health goals and preferred outcomes. Goals were categorised as Autonomy (information, security, and control) and Functioning (physical, psychosocial, and relief).

In choosing to attend for acute healthcare, participants sought relief from distressing symptoms and support to overcome limitations in physical and psychosocial function. Participants preferred varying degrees of active involvement in healthcare decision-making personally or with relatives and wished to be consulted on this. Living with frailty, having acute illness, and being in hospital were all associated with feeling vulnerable. Participants sought reassurance and support because they felt unsafe due to disabilities, physical or mental health problems, or their living environment.

### Relationships with existing knowledge

The earlier systematic review (which considered older people in general) had emphasised the need for system accessibility and explanations for inefficiency [15]. Conversely in this study, participants with frailty required explanations about illness severity and the possible consequences, and attention to functional limitations so that they could maintain their current activity level. While this study recruited from a single UK hospital, the sample broadly represented the diverse city population. Older people living with frailty in other European countries have also been reported to prioritise their health-related quality of life over mortality or waiting times [29, 30].

The systematic review had identified a need for reassurance to allay older people’s feelings of vulnerability, whereas this study identified a starker, daily insecurity attributed to potential deterioration. While older people in general feared losing function, participants living with frailty had already lost function and were concerned about the impact upon others. This loss of function has been described as the determining factor in life experiences for people as they become progressively frail [18, 31].

Similarities with outcomes from the previous review include symptom relief, involvement of relatives, and understanding decisions. Older people in general wished to be active decision-makers, whereas that was not always the case among these participants with frailty, who first wished to be asked about their preferred degree of involvement. Indeed, in a recent US survey, as many as one third of older people preferred to delegate healthcare decisions to professionals or their relatives [32]. Older people from East Asian (including Chinese) cultures have often been considered by relatives and clinicians to more willingly assume a role as passive recipients of care [33]. While we must acknowledge that our sampling and interviewing approach did not set out to test for such an effect, there was no observed difference in perspectives between participants of different ethnicities.

This framework of acute healthcare outcome goals has many similarities but also some important differences to previous models considering general healthcare settings. These differences might partly be related to rapid flow, interactions with multiple different healthcare providers and uncertainty in the face of health crises which are predominant in the acute setting. While themes of physical function, pain and symptom relief, and control in decision-making appear in Comprehensive Geriatric Assessment and International Consortium for Health Outcomes Measurement models, themes of loneliness, continence, and financial independence were not apparent as acute healthcare outcome goals, perhaps because participants did not consider the ED to be the appropriate setting to express such concerns [34, 35].

Interview participants were aware of the ED being busy, were mindful of their place in the queue, and were generally accepting of long waits to be seen. Participants often expressed concern at burdening the healthcare system or wasting professionals’ time. In crowded healthcare systems where service metrics prevail, there is a danger that time targets divert attention from patient comfort. Primary care literature has reported that people feel like they are on a conveyer belt of patients, and are often concerned that their problem will not be serious enough to warrant a doctor’s time [36]. Similar perceptions were apparent among participants in this study, who were often unsure of the ‘right time’ to access emergency care; some participants had suffered symptoms for long periods before seeking help. There is currently no validated mechanism to measure Patient-Reported Outcomes for older people with frailty receiving acute care, although such instruments are currently being developed [16, 37]. In the meantime, this framework reminds us of the importance of grounding our principles and practice in the needs of patients, which may not always be represented in service-level targets.

### Limitations

Interviews were conducted with patient participants from a single UK hospital. Purposive recruitment broadly represented the cultural and social backgrounds of the local city population. While the health and social conditions contributing to frailty are likely to be similar in other communities, the resulting perceptions and outcome goals may differ according to the social context.

Only one participant had Clinical Frailty Score 8. People with CFS 8 represent around one-seventh of ED patients who have frailty, and so were under-represented in this sample of twenty-two [38]. Similarly, fewer people living in residential care settings were recruited than we had anticipated. Those who we approached who had the most severe frailty often lacked consent or participation capacity due to advanced cognitive impairment, despite robust efforts to engage them via consultee processes.

Our involvement of accompanying relatives or familiar caregivers widened inclusion in a more diverse sample: perspectives were observed from some people with communication difficulties, including due to cognitive impairment, medical conditions, or language, where normally these participants might have been excluded from research. The presence of accompanying participants enhanced reflections and aided clearer expression. However, the involvement of other parties does introduce some limitations. Participants may have felt unable to express certain topics, including healthcare outcome goals, due to embarrassment or fear in the presence of their relative or caregiver. Unfortunately, any such topics would usually remain undisclosed in consultations with healthcare professionals, too.

Although the interviewer introduced themselves as a university researcher, it may have become evident that they were also medically trained. Effort was made to appear impartial, and participants were encouraged to share negative as well as positive perspectives. However, participants likely perceived a connection between the researcher and the clinical team, particularly when they were interviewed in hospital. This could be expected to have influenced the data, with participants less willing to share negative views about their care or professionals. The interviews were conducted by one single researcher. To mitigate for the effects of interviewer biases, transcript data were analysed by three researchers separately and then compared. Emerging themes were validated through searching for disconfirming cases during participant feedback in subsequent interviews and with lay research partners.

### Implications for research

The involvement as research partners of people living with frailty has previously been described as ‘unfeasible’, and researchers have often relied on people’s relatives and healthcare professionals [39]. Not only were we able to engage people with a range of levels of frailty in potentially complex discussions, but they had actively reflected upon their outcomes prior to the hospital attendance. Moreover in this study, older people were highly engaged and actively involved at every stage as lay research partners. Their involvement ensured that approaches were relevant and appropriate, and that findings remained firmly grounded in a patient voice.

### Implications for clinical practice

Feeling in control of health and healthcare and being informed and empowered to make decisions are related to the need to feel safe in place and routine. ‘Security’ as an outcome goal appears to be novel and corresponds to the uniquely unstable and uncertain health states older people have in the period leading to, during, and immediately following an acute, emergency, or urgent care episode.

While patients often seek to receive a diagnosis, emergency care professionals typically seek to rule-out life-threatening conditions, and therefore diagnoses may not be reached in this setting [40]. Participants found this uncertainty difficult to accept without explanation, expecting the tests and processes they underwent to provide the desired information. Interview participants often sought reassurance about the severity of their problem, which can be provided even in the absence of a diagnosis.

Consultations could support patients’ requirements by using shared decision-making models [41]. We had approached this study with an assumption that shared decision-making was always an aspirational goal. However, not all interview participants wished to be active decision-makers. Professionals should elicit preferences regarding the nature and depth of information and the extent of involvement in decision-making [32, 42].

Many participants felt concerned about burdening the healthcare system or wasting professionals’ time. They had often carefully considered accessing acute care, after exhausting community-based options or contacting relatives as if to seek validation [43]. There is a risk that crowded systems becoming process-oriented at the expense of being person-centred, thus exacerbating concerns of legitimacy. Concerns may be allayed through affirmation during consultations [36, 44]

## Conclusions

Acute healthcare outcome goals among older people with frailty can be categorised as ‘Autonomy’ (information, control, and security) and ‘Functioning’ (physical, psychosocial, and relief). The novel sub-theme ‘security’, comprising feeling safe in living place, routine, and health, was common to most interview participants. Healthcare professionals should be mindful of the insecurity which people with frailty live with day-to-day, and thus appreciate their need for reassurance around likely illness and recovery trajectories. Patients will have probably reflected on their health and outcome goals and may have delayed seeking healthcare for fear of being a burden or of not recovering. It should be remembered that frailty reflects the heterogeneity of the older population: outcome goals and decision-making preferences should be explored and ascertained on an individual basis. However, this framework can be considered and discussed with older people with frailty in the acute setting to aid elicitation of outcome goals and consequent holistic management.

## Data Availability

The code structure is available from the corresponding author on reasonable request.

## Appendix 1: interview topic guide

Authors’ note: the interviews were semi-structured using the topic guide questions as prompts only. The interviews did not follow a rigid format. Discussion flowed as guided by the conversation.

Can you tell me about your recent experience of needing to attend the Emergency Department?

- Explore events leading to attendance to aid recall.
- What made you choose to go to the ED?
- Did you need help to get there? Who did you call? Was it convenient and accessible?
- What were your worries about going to hospital?
- What treatments did you hope the doctors, nurses and therapists would give you?
- What sort of setting did you want to be looked after in? Room, furniture, decoration.
- What were you hoping they might explain to you?
- Who did you want to be involved in your care?

Can you tell me about the care you received?

- What treatments or help did you receive?
- What has changed as a result of your time in the Emergency Department?
- What was good? What could have been better?
- What made a difference, or would have made a difference?

**Anything not covered?** Is there anything that we haven’t covered in the interview that you think we should know or think about?

Researcher post-interview comments:

## Abbreviations

UK: United Kingdom
ED: Emergency Department
CFS: Clinical Frailty Score
US: United States

## References

[1] Morley C, Unwin M, Peterson GM, Stankovich J, Kinsman L (2018). Emergency department crowding: A systematic review of causes, consequences and solutions. PloS one.

[2] Singler K, Christ M, Sieber C, Gosch M, Heppner HJ (2011). Geriatrische Patienten in Notaufnahme und Intensivmedizin. Der Internist.

[3] Keeble E, Roberts HC, Williams CD, Van Oppen J, Conroy SP (2019). Outcomes of hospital admissions among frail older people: a 2-year cohort study. Br J Gen Pract.

[4] Considine J, Berry D, Rasmussen B, Hutchinson AM, Rawson H, Jordan P (2021). Impact of emergency department length of stay on anxiety and comfort in older people. International Emergency Nursing..

[5] Fletcher AE, Price GM, Ng ESW, Stirling SL, Bulpitt CJ, Breeze E, et al. Population-based multidimensional assessment of older people in UK general practice: a cluster-randomised factorial trial. Lancet. 364:9446, 1667–77. 10.1016/S0140-6736(04)17353-4 15530627

[6] Rockwood K, Mitnitski A (2011). Frailty Defined by Deficit Accumulation and Geriatric Medicine Defined by Frailty. Clin Geriatric Med.

[7] Rockwood K, Song X, MacKnight C, Bergman H, Hogan DB, McDowell I (2005). A global clinical measure of fitness and frailty in elderly people. CMAJ.

[8] Elliott A, Taub N, Banerjee J, Aijaz F, Jones W, Teece L, et al. Does the Clinical Frailty Scale at Triage Predict Outcomes From Emergency Care for Older People? Ann Emerg Med. 2020. 10.1016/j.annemergmed.2020.09.006 33328147

[9] Elliott A, Phelps K, Regen E, Conroy SP (2017). Identifying frailty in the Emergency Department—feasibility study. Age and Ageing.

[10] Conroy S, Carpenter C, Banerjee J. Silver Book II. London, UK. Brit Geriatrics Soc. 2021.

[11] Ellis G, Whitehead M, O'Neill D, Robinson D, Langhorne P (2011). Comprehensive geriatric assessment for older adults admitted to hospital. Cochrane Database Syst Rev.

[12] Institute for Healthcare Improvement. Age-Friendly Health Systems: Guide to Using the 4Ms in the Care of Older Adults. 2020.

[13] Naik AD, Dindo LN, Van Liew JR, Hundt NE, Vo L, Hernandez-Bigos K (2018). Development of a clinically feasible process for identifying individual health priorities. J Am Geriatr Soc.

[14] van Oppen JD, Valderas JM, Mackintosh NJ, Conroy SP. Patient-reported outcome and experience measures in geriatric emergency medicine. Z Gerontol Geriatr. 2020. 10.1007/s00391-020-01777-4 32939573

[15] van Oppen JD, Keillor L, Mitchell A, Coats TJ, Conroy SP (2019). What older people want from emergency care: a systematic review. Emerg Med J.

[16] Melady D (2018). Geriatric emergency medicine: Research priorities to respond to "The Silver Boom". CJEM.

[17] van Munster BC, Boot GG, Festen SF, de Rooij SE. Goals and outcomes of hospitalised older people: does the current hospital care match the needs of older people? Intern Med J. 2021. 10.1111/imj.15508 PMC9314846 34490694

[18] Cluley V, Martin G, Radnor Z, Banerjee J. Frailty as biographical disruption. Sociol Health Illn 2021;n/a(n/a). 10.1111/1467-9566.13269 33969903

[19] Fusch P, Ness L (2015). Are We There Yet? Data Saturation in Qualitative Research. Qual Rep.

[20] Hennink MM, Kaiser BN, Marconi VC (2016). Code Saturation Versus Meaning Saturation: How Many Interviews Are Enough?. Qual Health Res.

[21] Bengtsson M (2016). How to plan and perform a qualitative study using content analysis. Nursing Plus Open.

[22] QSR International Pty Ltd. NVivo. 11 ed2015.

[23] Merriam SB, Tisdell EJ (2015). Qualitative Research : A Guide to Design and Implementation.

[24] Glaser BG (1965). The Constant Comparative Method of Qualitative Analysis. Social Problems.

[25] Glaser BG, Strauss A (1967). The discovery of grounded theory: Strategies for qualitative research.

[26] Glaser BG, Holton JA. Staying Open: The use of theoretical codes in grounded theory. Grounded Theory. Review. 2005;5(1).

[27] Tong A, Sainsbury P, Craig J (2007). Consolidated criteria for reporting qualitative research (COREQ): a 32-item checklist for interviews and focus groups. Int J Qual Health Care.

[28] Preston L, van Oppen J, Conroy S, Ablard S, Buckley Woods H, Mason S. Improving outcomes for older people in the emergency department: a review of reviews. Emerg Med J. 2020. 10.1136/emermed-2020-209514 33106287

[29] Olde Rikkert MGM, van der Wees PJ, Schoon Y, Westert GP (2018). Using Patient Reported Outcomes Measures to Promote Integrated Care. Int J Integr Care.

[30] Heeß A, Meyer AM, Becker I, Noetzel N, Verleysdonk J, Rarek M, et al. The prognostic fingerprint of quality of life in older inpatients : Relationship to geriatric syndromes' and resources' profile. Z Gerontol Geriatr. 2021. 10.1007/s00391-021-01978-5 34617144

[31] Søvde BE, Sandvoll AM, Natvik E, Drageset J. In the borderland of the body: How home-dwelling older people experience frailty. Scand J Caring Sci 2021;n/a(n/a). 10.1111/scs.12984 33939195

[32] Wolff JL, Boyd CM (2015). A Look at Person-Centered and Family-Centered Care Among Older Adults: Results from a National Survey. J Gen Intern Med.

[33] Woo J (2020). The Myth of Filial Piety as a Pillar for Care of Older Adults among Chinese Populations. Adv Geriatric Med Res.

[34] Akpan A, Roberts C, Bandeen-Roche K, Batty B, Bausewein C, Bell D (2018). Standard set of health outcome measures for older persons. BMC Geriatr.

[35] Conroy SP, Bardsley M, Smith P, Neuburger J, Keeble E, Arora S (2019). Comprehensive geriatric assessment for frail older people in acute hospitals: the HoW-CGA mixed-methods study. Health Serv Deliv Res.

[36] Llanwarne N, Newbould J, Burt J, Campbell JL, Roland M (2017). Wasting the doctor's time? A video-elicitation interview study with patients in primary care. Soc Sci Med.

[37] van Oppen JD. Testing and validation of a Patient-Reported Outcome Measure for Older People with frailty and Acute Care needs (PROM-OPAC). Protocolsio. 2021. 10.1186/s41687-024-00796-8 PMC11484973 39414637

[38] Sablerolles RSG, Lafeber M, van Kempen JAL, van de Loo BPA, Boersma E, Rietdijk WJR (2021). Association between Clinical Frailty Scale score and hospital mortality in adult patients with COVID-19 (COMET): an international, multicentre, retrospective, observational cohort study. Lancet Healthy Longevity.

[39] Hansen TK, Jensen AL, Damsgaard EM, Rubak TMM, Jensen MEJ, Gregersen M (2021). Involving frail older patients in identifying outcome measures for transitional care—a feasibility study. Res Involv Engagem.

[40] Hagerty SF, Burke RC, Isbell LM, Barasz K, Smulowitz P. Patient Perceptions of Diagnostic Certainty at Discharge and Patient Satisfaction in the ED. Academic Emergency Medicine. 2021;n/a(n/a). 10.1111/acem.14262 33830597

[41] Elwyn G, Frosch D, Thomson R, Joseph-Williams N, Lloyd A, Kinnersley P (2012). Shared decision making: a model for clinical practice. J Gen Intern Med.

[42] Chewning B, Bylund CL, Shah B, Arora NK, Gueguen JA, Makoul G (2012). Patient preferences for shared decisions: A systematic review. Patient Educ Counseling.

[43] Hunter C, Chew-Graham C, Langer S, Stenhoff A, Drinkwater J, Guthrie E (2013). A qualitative study of patient choices in using emergency health care for long-term conditions: The importance of candidacy and recursivity. Patient Education and Counseling..

[44] Dixon-Woods M, Cavers D, Agarwal S, Annandale E, Arthur A, Harvey J (2006). Conducting a critical interpretive synthesis of the literature on access to healthcare by vulnerable groups. BMC Med Res Methodol.

